# Machine learning-assisted Raman spectroscopy for non-destructive analysis of crude palm oil quality

**DOI:** 10.1038/s41538-025-00688-1

**Published:** 2026-01-14

**Authors:** Selorm Yao-Say Solomon Adade, Akwasi Akomeah Agyekum, Xorlali Nunekpeku, Nana Adwoa Nkuma Johnson, John-Nelson Ekumah, Bridget Ama Kwadzokpui, Hao Lin, Huanhuan Li, Quansheng Chen

**Affiliations:** 1 College of Ocean Food and Biological Engineering, Jimei University, Xiamen, PR China; 2 Centre for Agribusiness Development and Mechanization in Africa (CADMA AgriSolutions), Ho, Ghana; 3 Department of Nutrition and Dietetics, Ho Teaching Hospital, Ho, Ghana; 4 Nutrition Research Centre, Ghana Atomic Energy Commission, Accra, Ghana; 5 School of Food and Biological Engineering, Jiangsu University, Zhenjiang, PR China

**Keywords:** Chemistry, Engineering, Mathematics and computing

## Abstract

Quality assessment of crude palm oil remains a critical challenge globally, particularly in resource-poor areas where traditional methods are time-consuming and destructive. This study explores machine learning-assisted Raman spectroscopy for non-destructive assessment of peroxide value (PV) and iodine value (IV) in palm oil. Raman spectra were collected from 200 samples from five Ghanaian markets, with second derivative preprocessing significantly enhancing feature resolution. Twelve predictive models were developed by combining three variable selection algorithms (CARS, GA, UVE) with three regression methods (PLS, SVM, RF). The genetic algorithm-random forest (GA-RF) model demonstrated exceptional prediction accuracy for both PV (Rp = 0.9831, RPD = 7.7397) and IV (Rp = 0.9752, RPD = 6.3927). Key spectral regions associated with unsaturation (1287-1657 cm⁻¹) and oxidation (1748-1840 cm⁻¹) were identified as crucial predictors. This approach enables rapid, non-destructive quality assessment with potential applications throughout the palm oil value chain.

## Introduction

Crude palm oil (CPO) quality assessment is a critical bottleneck in developing economies, where traditional analytical methods create significant operational inefficiencies. In Ghana’s palm oil industry, which produces approximately 300,000 metric tons annually^1^, the separate determination of peroxide value (PV) and iodine value (IV) requires multiple time-consuming procedures that are particularly problematic in resource-constrained settings. The Wijs titration method for IV determination and the acetic acid-chloroform extraction for PV measurement each take several hours. It requires hazardous reagents, forcing many smallholder operations to rely on subjective quality evaluations^2^. This sequential analytical approach not only increases costs and environmental impact but also prevents the real-time quality control essential for optimizing processing operations and ensuring export compliance^3^.

The fundamental challenge lies in developing a single analytical method capable of simultaneously determining both oxidative stability (PV) and the degree of unsaturation (IV) in crude palm oil matrices. Crude palm oil poses significantly more complex analytical challenges than refined oils due to matrix interference from carotenoids, chlorophylls, and free fatty acids^4^. Specifically, β-carotene concentrations reaching 500-700 ppm generate strong fluorescence backgrounds that mask critical Raman bands in the 1600–1700 cm^−1^ region where C=C and C=O stretching vibrations provide essential information for IV and PV determination^5^. Additionally, free fatty acids (typically 3–5% in crude palm oil) introduce spectral complexity by generating additional carbonyl signals that overlap with oxidation product peaks, requiring multivariate deconvolution to distinguish between inherent composition and degradation-related signals^4^.

Recent advances in machine learning-assisted spectroscopy have shown exceptional potential for handling complex analytical challenges, with deep learning approaches achieving coefficient of determination (R^2^) values exceeding 0.995 for various oil parameters^6^. However, a critical research gap exists in the simultaneous prediction of iodine and peroxide values using pure Raman spectroscopy for crude palm oil analysis. Existing studies have predominantly focused on the determination of individual parameters^7,8^. In contrast, the few studies that have attempted simultaneous determination of quality parameters have either focused on different parameter combinations or employed refined edible oil systems that lack the matrix complexity challenges inherent in crude palm oil analysis^7^. Achieving accurate simultaneous prediction of both parameters is particularly challenging given the matrix interference from carotenoids and free fatty acids, which complicates even individual parameter determinations.

This intersection of crude palm oil’s matrix complexity and the need to simultaneously determine IV and PV creates a unique analytical challenge that has not been systematically addressed. Simultaneous prediction offers significant methodological advantages beyond mere convenience, as these parameters exhibit inverse correlations that provide opportunities for internal validation of measurement accuracy^9^. Furthermore, real-time processing optimization requires rapid assessment of both parameters to prevent quality degradation during storage and transport operations. Cross-parameter validation from single spectral measurements also enables uncertainty quantification, potentially improving analytical reliability compared to sequential individual determinations^10^.

This study addresses this gap through the development and validation of machine learning-assisted Raman spectroscopy for concurrent IV and PV determination in crude palm oil samples. The research specifically investigates: (1) the capability of advanced machine learning algorithms to extract simultaneous quality parameter information from complex crude palm oil Raman spectra, (2) the comparative performance of different ML approaches against traditional chemometric methods for this specific dual-prediction challenge, and (3) the method validation and operational feasibility through assessment of calibration model robustness, measurement reproducibility, and comparison with standard reference methods for accuracy verification. By demonstrating successful simultaneous prediction from single spectral measurements, this research establishes a methodological foundation for more efficient, cost-effective, and environmentally sustainable palm oil quality assessment practices in developing economies.

## Results

### Reference measurement results for PV and IV

The results in Table 1 reveal important patterns in the physicochemical properties of palm oil across five different sources (AGB, DOM, KAN, MAD, and MAK). The peroxide values ranged from 4.29 to 11.45 meq O_2_/kg, with KAN samples exhibiting the highest mean peroxide value (8.23 ± 1.65 meq O_2_/kg) and AGB samples showing the lowest (6.79 ± 2.08 meq O_2_/kg). Most values fell within the critical threshold of 10 meq/kg established by international standards, indicating acceptable oxidative stability^11^. These findings align with Agbaire^12^ and Enyoh et al.^4^, who reported similar values for palm oil samples from Nigerian markets. Notable variability in peroxide values was observed, particularly in MAK samples, which displayed the highest coefficient of variation (0.307), suggesting inconsistent oxidative stability despite a moderate mean value (7.79 ± 2.39 meq O_2_/kg).

**Table 1:** Descriptive statistics for peroxide and iodine values in crude palm oil

Parameter	Palm oil samples source	Range	(Mean ± SD)	Coefficient of Variation (SD/Mean)
Peroxide value (meq O_2_/kg)	AGB	4.66–11.38	6.79 ± 2.08^a^	0.306
	DOM	4.36–10.77	7.58 ± 1.86^b^	0.245
	KAN	4.48–10.65	8.23 ± 1.65^c^	0.201
	MAD	4.29–11.22	8.10 ± 1.87 ^d^	0.231
	MAK	4.57–11.45	7.79 ± 2.39^e^	0.307
Iodine value (g I_2_/100 g)	AGB	42.69–48.31	45.57 ± 1.62^a^	0.036
	DOM	43.72–48.49	46.44 ± 1.37^b^	0.030
	KAN	41.78–48.63	46.51 ± 1.98^c^	0.043
	MAD	40.89–46.19	45.04 ± 2.00 ^d^	0.044
	MAK	45.67–48.69	47.24 ± 0.91^e^	0.019

Sample means along the same column with different superscript alphabets are significantly different at p < 0.05.

*AGB* Agbogbloshie, *DOM* Dome, *KAN* Kaneshie, *MAD* Madina, and *MAK* Makola.

Similarly, the iodine values were consistently lower than the standard range of 50.0-55.0 g I₂/100 g across all sources. MAK samples demonstrated the highest mean iodine value (47.24 ± 0.91 g I₂/100 g) and the lowest variability (CV = 0.019), indicating a remarkably uniform fatty acid composition despite highly variable peroxide values. This pattern mirrors observations by Ngunoon et al.^13^ and MacArthur et al.^3^, who similarly found low values in palm oil samples from Nigerian and Ghanaian markets.

The inverse relationship between peroxide value variability and iodine value consistency in MAK samples suggests that oxidative degradation processes may operate independently of the initial unsaturation profile. This finding aligns with MacArthur et al.^3^, who observed that quality parameters in palm oil samples from different regions varied independently, suggesting that post-harvest handling likely exerts a greater influence on quality than intrinsic compositional factors.

The consistently low iodine values across all samples have important implications for oil quality and application in domestic and industrial contexts. While a lower degree of unsaturation typically indicates greater oxidative stability, it may also suggest possible adulteration with other, more saturated fats or oils, as cautioned by Ngunoon et al.^13^ in their study of palm oil quality in Nigerian markets. Additionally, these low iodine values might result from variations in processing techniques employed by smallholder producers, as palm oil production in Ghana is dominated by traditional processing methods that may influence the final fatty acid composition of the oil^2^. Understanding these relationships between processing methods, fatty acid composition, and resulting physicochemical properties is crucial for establishing appropriate quality standards and improving palm oil production practices to ensure consistent quality across diverse sources.

### Examination of Raman spectra

**Fig. 1:**
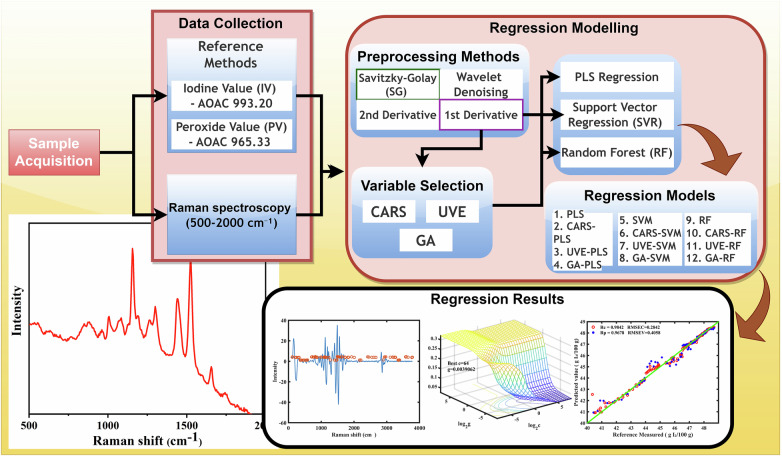
Schematic workflow of machine learning-assisted Raman spectroscopy for non-destructive quality assessment of crude palm oil. This figure illustrates the complete workflow for the non-destructive quality assessment of crude palm oil using machine learning-assisted Raman spectroscopy. The process begins with sample acquisition, followed by data collection using standard reference methods, including the determination of iodine (IV) and peroxide (PV) values as per AOAC protocols. The Raman spectral data (500–2000 cm⁻¹) is then preprocessed using various techniques, such as Savitzky-Golay smoothing, wavelet denoising, and derivative transformations (1st and 2nd derivatives). The next step involves variable selection using methods such as CARS, UVE, and GA, which are used to optimize the data. Subsequently, regression modeling is applied using various machine learning algorithms, including PLS, CARS-PLS, UVE-PLS, GA-PLS, SVM, and Random Forest, to predict the quality parameters of crude palm oil. The regression results section displays the correlation between the predicted values and reference measurements, with a strong predictive ability indicated by the high correlation coefficients (Rc and Rp).

Raman spectral profiles of crude palm oil from major markets (AGB, DOM, KAN, MAD, and MAK) reveal distinct vibrational modes essential for predicting iodine value (IV) and peroxide value (PV). As shown in Fig. 1A, the raw spectra (500–2000 cm^−1^) exhibit features associated with unsaturation and oxidation but suffer from baseline variations and varying peak intensities. Baseline correction and normalization (Fig. 1C) address these issues, enhancing the clarity of spectral features and enabling better assessment of unsaturation levels. A closer look at a representative spectrum (Fig. 1B, raw; Fig. 1D, processed) highlights peaks at 1156, 1442, 1524, and 1657 cm^−1^, attributed to C–H bending and C=C stretching vibrations. The peak at 1748 cm^−1^, associated with C=O stretching in oxidation-related functional groups like peroxides, is particularly significant. Peaks at 1287, 1602, and 1657 cm^−1^, arising from cis =C–H bending and C=C stretching, are especially relevant to IV determination, as they reflect the degree of unsaturation in fatty acids. These findings align with previous studies linking Raman spectra to oil quality^14,15^.

The vibrational markers listed in Table S1 allow rapid, non-destructive assessment of unsaturation (IV) and oxidation (PV) in crude palm oil. Unlike labor-intensive chemical titration methods, Raman spectroscopy provides real-time sensitivity to molecular composition and oxidation-state changes. This is crucial for evaluating oil stability, as oxidation accelerates in the presence of air, heat, and light. Monitoring peak intensities enables manufacturers to determine when oils approach quality limits for consumption or processing, offering a practical and efficient alternative for quality control^14–16^.

### Preprocessing results

We preprocessed the Raman spectra for PV and IV using four distinct methods: 1st Der, 2nd Der, WD, and SG, with raw spectra as a control (Table S2). For peroxide value prediction, the 2nd Der preprocessing algorithm demonstrated marked superiority, with the highest correlation coefficients in both calibration (Rc = 0.9597) and prediction sets (Rp = 0.9481), and a notably high RPD value of 4.8935. This superior performance results from the second derivative’s ability to effectively eliminate baseline effects while enhancing spectral resolution, particularly important for complex palm oil matrices where overlapping bands are common^17^. Despite having a slightly higher RMSEC (0.5154 meq O₂/kg) than the WD method (0.4297 meq O₂/kg), the 2nd Der approach demonstrated significantly better predictive capacity, as evidenced by its substantially higher RPD (4.8935 vs. 3.0606).

For iodine value determination, the 2nd Der approach again showed exceptional performance with the highest correlation coefficient in the calibration set (Rc = 0.9692), an excellent prediction correlation (Rp = 0.9400), the lowest RMSEP of 0.5002 g I₂/100 g, and an impressive RPD of 3.9139. The WD method, while showing good performance (Rc = 0.9546, Rp = 0.9325), was notably inferior in predictive capability with a higher RMSEP (0.5951 g I₂/100 g vs. 0.5002 g I₂/100 g) and lower RPD (3.8336 vs. 3.9139). The 1st Der preprocessing yielded the poorest results for IV prediction (RMSEP = 0.8195 g I₂/100 g, RPD = 2.7613), likely due to its reduced ability to resolve overlapping spectral features associated with unsaturated fatty acids, which strongly influence iodine value. The raw spectra approach, although showing reasonable calibration performance, demonstrated inadequate predictive capacity for both parameters, with RPD values of 2.7880 (PV) and 3.4346 (IV), highlighting the need for appropriate preprocessing for robust model development.

The second-derivative preprocessing method provided superior results for predicting peroxide and iodine values due to enhanced resolution of overlapping spectral bands, reduced baseline variations, and improved signal-to-noise ratio when combined with optimal principal component selection^18–20^. This optimized technique was selected as the input for all subsequent analyses, resulting in more accurate and robust predictive models for rapid, non-destructive palm oil quality assessment^21^.

### Results of variable selection and chemometrics

**Fig. 2:**
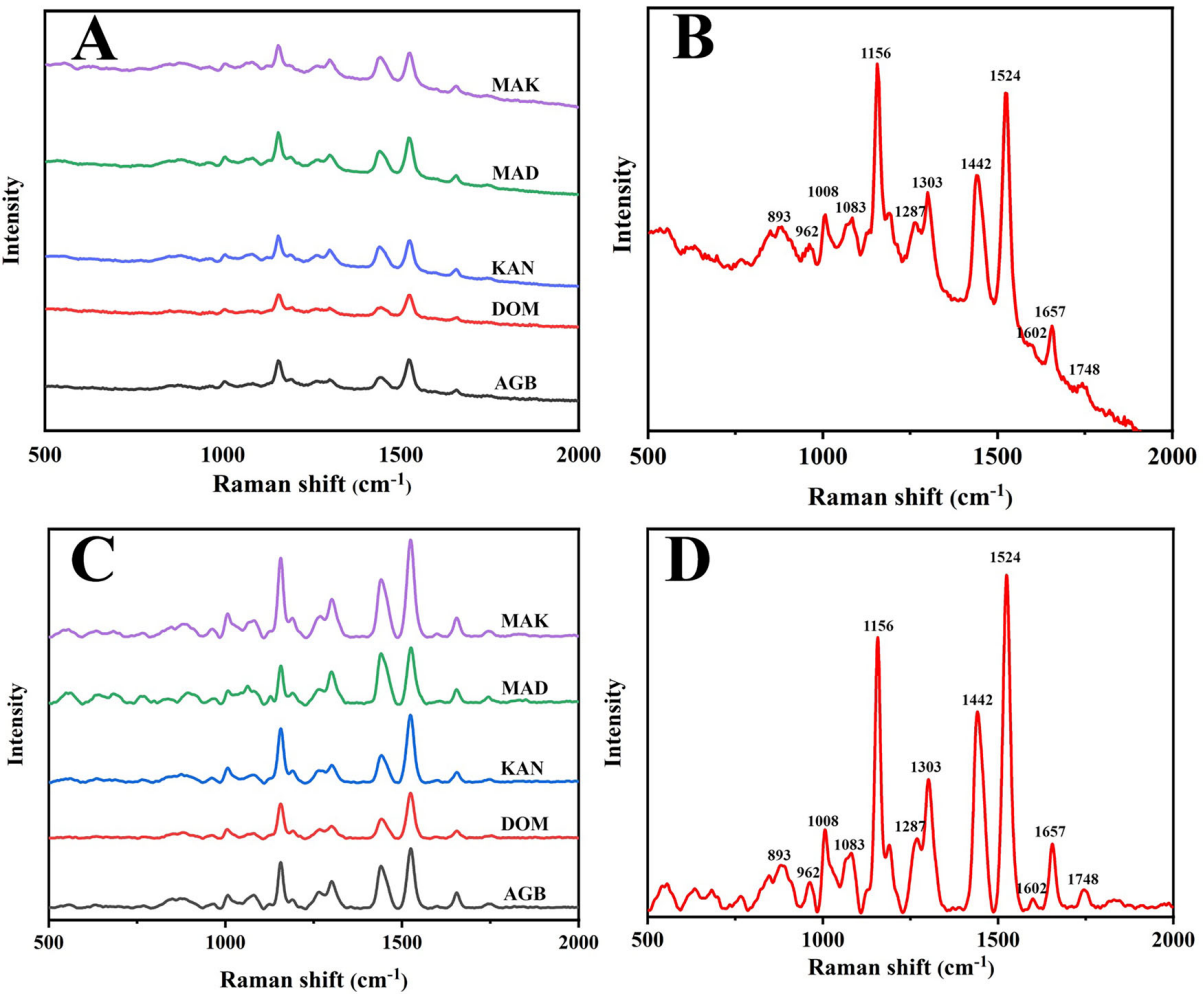
Raw and baseline corrected + normalized Raman spectra of crude palm oil samples from major markets. **A** Raw Raman spectra of crudeAQ9 palm oil samples from major markets (AGB, DOM, KAN, MAD, and MAK). Each spectrum is vertically offset for clarity. **B** A typical raw Raman spectrum of crude palm oil, highlighting key spectral peaks. **C** Baseline-corrected and normalized spectra of the same crude palm oil samples, where preprocessing has been applied to enhance the spectral features. **D** Baseline-corrected and normalized spectrum of a typical crude palm oil sample, with labeled peaks indicating key Raman shift values (e.g., 893 cm^−1^, 1156 cm^−1^, 1657 cm^−1^), which are characteristic of palm oil’s molecular composition.

We applied three variable selection algorithms—CARS, UVE, and GA—to the preprocessed Raman spectra to identify the most informative features for predicting peroxide value (PV) and iodine value (IV). For PV, the CARS algorithm (Fig. 2A–E) progressively eliminated redundant variables, achieving the lowest RMSECV around the 20th iteration when 104 variables were retained, primarily between 1000 and 1500 cm^−1^. UVE (Fig. 2F–J) provided the greatest dimensionality reduction, retaining 66 variables (6.4%) concentrated around unsaturated bond and carbonyl regions associated with oxidation products. GA (Fig. 2K–O) selected 148 variables in a balanced manner through iterative evolutionary optimization, highlighting oxidation-related C=O stretching vibrations near 1748 cm^−1^. Together, these results confirm that the three algorithms effectively reduced redundancy while preserving key chemical information relevant to oxidation dynamics in crude palm oil.

**Fig. 3:**
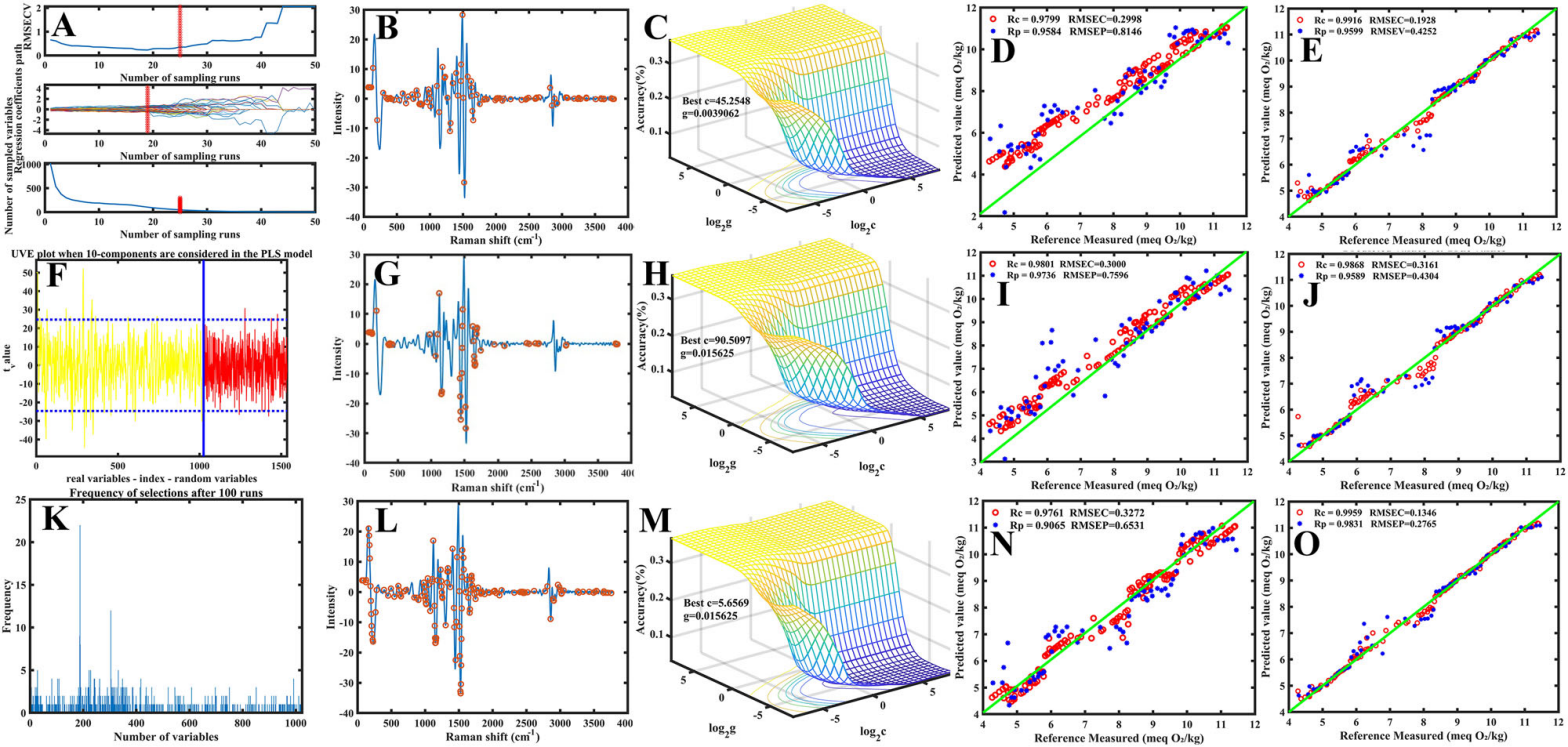
Results of variable selection algorithms and machine learning models for peroxide value (IV) prediction in crude palm oil using Raman spectroscopy. CARS variable selection results: **A** variable selection process showing RMSECV values and the number of retained variables, **B** distribution of selected variables in the full Raman spectrum, **C** 3D response surface for SVM hyperparameter optimization, **D** scatter plot of CARS-SVM model predictions versus reference values, **E** scatter plot of CARS-RF model predictions versus reference values. UVE variable selection results: **F** reliability plot distinguishing relevant variables (yellow) from random variables (red), **G** distribution of selected variables, **H** 3D response surface for SVM hyperparameter optimization, **I** UVE-SVM prediction scatter plot, **J** UVE-RF prediction scatter plot. GA variable selection results: **K** frequency distribution of selected variables, **L** distribution of selected variables in the spectrum, **M** 3D response surface for SVM hyperparameter optimization, **N** GA-SVM prediction scatter plot, **O** GA-RF prediction scatter plot. Across all three variable selection strategies, the majority of selected variables are concentrated in chemically meaningful Raman regions associated with lipid unsaturation and oxidation, particularly 1287–1657 cm^−1^ (C–H bending and C=C stretching) and the carbonyl-related region around ~1748 cm^−1^, which underpin peroxide value prediction.

For IV prediction, a similar workflow was applied, allowing direct comparison between oxidation- and unsaturation-related spectral dependencies. The CARS algorithm (Fig. 3A–E) retained 91 variables, with a slightly broader distribution toward the 1600–1650 cm^−1^ region, reflecting stronger C=C contributions. UVE (Fig. 3F–J) further reduced the variable set to 45 (4.4%), emphasizing cis =C–H bending and C=C stretching bands characteristic of unsaturated fatty acids. GA (Fig. 3K–O) identified 140 variables, with a greater focus on the 1657 cm^−1^ region associated with unsaturation. Compared with PV, the IV models relied more heavily on spectral features associated with double-bond vibrations, demonstrating that each algorithm dynamically adjusted its variable weighting based on the underlying chemical property.

### Results of partial least squares (PLS) regression

After variable selection identified relevant spectral features, partial least squares (PLS) regression was employed. This statistical technique helped establish quantitative relationships between the selected variables and the quality parameters of crude palm oil. The models developed from spectral regions of CARS, UVE, and GA were compared with standard PLS using the full spectral dataset. Table 1 summarizes the models’ calibration and prediction metrics for peroxide value (PV) and iodine value (IV). For PV prediction, variable selection significantly improved computational efficiency. Standard PLS, using all 1024 variables, performed well (Rp = 0.9597, RMSEP = 0.6367 meq O₂/kg, RPD = 4.8935). However, optimized models achieved similar accuracy with fewer variables. CARS-PLS, utilizing 104 variables, achieved the highest calibration correlation (Rc = 0.9794) but lower prediction reliability (RPD = 2.3079). UVE-PLS, retaining only 66 variables, demonstrated excellent predictive performance (Rp = 0.9613, RPD = 3.6138), with a 93.6% reduction in dimensionality. GA-PLS achieved balanced results with 148 variables (Rp = 0.9546, RPD = 3.0003) (Table 2).

**Table 2:** Predictive performance of chemometric models with variable selection algorithms for peroxide and iodine value prediction in crude palm oil

Parameter	Models	PCs	Number of variables	Calibration set n = 133		Prediction set n = 67		RPD
				Rc	RMSEC	Rp	RMSEP	
Peroxide value (meq O_2_/kg)	PLS	6	1024	0.9597	0.5154	0.9481	0.6367	4.8935
	CARS-PLS	9	104	0.9794	0.4701	0.9557	0.5649	2.3079
	UVE-PLS	6	66	0.9705	0.5104	0.9613	0.5186	3.6138
	GA-PLS	6	148	0.9641	0.5603	0.9471	0.6824	3.0003
Iodine value (g I_2_/100 g)	PLS	6	1024	0.9692	0.3565	0.9400	0.5002	3.9139
	CARS-PLS	8	91	0.9687	0.3161	0.9483	0.5340	2.3208
	UVE-PLS	7	45	0.9724	0.5266	0.9650	0.6203	3.7535
	GA-PLS	6	140	0.9647	0.5945	0.9327	0.8678	2.7623

*n* number of samples, *PCs* principal components, *PLS* partial least squares, *RPD* residual predicted deviation, *Rc* correlation coefficient for the calibration set, *RMSECV* root mean square error of cross-validation, *Rp* correlation coefficient for the prediction set, *RMSEP* root mean square error of prediction, *UVE-PLS* uninformative variable elimination partial least squares, *CARS-PLS* competitive adaptive reweighted sampling partial least squares, *GA-PLS* genetic algorithm partial least squares.

For IV prediction, UVE-PLS excelled with just 45 variables, delivering the highest prediction correlation (Rp = 0.9650) and reliability (RPD = 3.7535). GA-PLS, using 140 variables, achieved the lowest prediction error (RMSEP = 0.8678 g I₂/100 g, RPD = 2.7623). CARS-PLS, utilizing 91 variables, showed weaker prediction statistics (Rp = 0.9508, RPD = 2.3208). According to Nunekpeku et al.^22^, RPD values above 3.0, including standard PLS, UVE-PLS, and GA-PLS for PV, and standard PLS and UVE-PLS for IV, demonstrated analytical-grade performance. These findings highlight the effectiveness of variable selection in identifying chemically relevant spectral regions while enhancing efficiency.

### Results of machine learning tools

We next employed SVM and RF algorithms to predict the PV and IV in CPO, applying these models to both the full spectral data comprising 1024 variables and the optimized variable subsets identified through prior selection methods. For PV prediction using full spectral data, RF outperformed SVM, achieving superior calibration (Rc = 0.9833) and prediction (Rp = 0.9802, RMSEP = 0.2735 meq O₂/kg, RPD = 6.2240). In contrast, SVM showed lower reliability (Rp = 0.9439, RMSEP = 0.8715 meq O₂/kg, RPD = 2.1877). Similarly, for IV prediction, RF achieved higher prediction accuracy (Rp = 0.9725, RMSEP = 0.3748 g I₂/100 g, RPD = 6.0727) than SVM (Rp = 0.9559, RMSEP = 0.9236 g I₂/100 g, RPD = 2.3871). These results demonstrate RF’s ability to provide robust predictions even when processing the full spectral dataset.

Hyperparameter optimization was critical for improving SVM performance. Using 3D response surface plots (e.g., Fig. 2C, H, M for PV; Fig. 3C, H, M for IV), the penalty parameter (C) and kernel parameter (γ) were fine-tuned. For CARS-SVM models, optimal configurations were identified at C = 64.2548 and γ = 0.037062 for PV and C = 64, γ = 0.037062 for IV. Similar optimizations were conducted for UVE-SVM and GA-SVM models, achieving RMSECV values of 0.2997 meq O₂/kg, 0.3000 meq O₂/kg, and 0.3272 meq O₂/kg for PV, and 0.3306 g I₂/100 g, 0.4545 g I₂/100 g, and 0.3367 g I₂/100 g for IV, respectively. Despite these efforts, SVM models consistently underperformed RF, even after hyperparameter optimization, as reported by Zulfiqar et al.^23^ and Syarif and Wills^24^.

The performance gap widened when we applied models to optimized variable subsets. For PV prediction, GA-RF achieved exceptional results, with the highest calibration correlation (Rc = 0.9959), minimal calibration error (RMSEC = 0.1346 meq O₂/kg), and strong prediction metrics (Rp = 0.9831, RMSEP = 0.2765 meq O₂/kg, RPD = 7.7397). This exceeded the commonly accepted threshold for excellent predictive models (RPD > 3). Conversely, the GA-SVM model improved over standard SVM but still lagged behind RF (Rc = 0.9761, RMSEC = 0.3272 meq O₂/kg, Rp = 0.9065, RMSEP = 0.6531 meq O₂/kg, RPD = 3.0566). For IV prediction, GA-RF similarly excelled (Rc = 0.9947, RMSEC = 0.1638 g I₂/100 g, Rp = 0.9752, RMSEP = 0.3561 g I₂/100 g, RPD = 6.3927), while GA-SVM showed weaker performance (Rc = 0.9787, RMSEC = 0.3367 g I₂/100 g, Rp = 0.9030, RMSEP = 0.7103 g I₂/100 g, RPD = 3.0234). Scatter plots (e.g., Fig. 2D vs. 3E, and Fig. 3D vs. 4E) confirm RF’s superior predictive alignment compared to SVM. These results are consistent with those of Adugna et al.^25^, who reported superior performance of RF models over SVMs.

RF’s superior performance results from its ensemble learning approach, which constructs multiple decision trees and averages their predictions, effectively mitigating overfitting^26^, a common challenge in spectroscopic data analysis, where the number of variables often exceeds the number of samples^27^. Bootstrap sampling and random feature selection create diverse trees that collectively contribute to robust predictions, making RF particularly effective for handling complex, non-linear relationships in spectroscopic data^28^. Furthermore, RF demonstrates remarkable robustness against common spectroscopic challenges, including baseline variations, minor peak shifts, and fluctuations in signal-to-noise ratio—particularly relevant in complex biological matrices such as crude palm oil^29,30^. Its consistently high RPD values (4.9724–7.7397 for PV, 4.3962–6.3927 for IV) underscore its suitability for quality control and regulatory applications in the palm oil industry^31,32^. These findings, consistent with recent applications of RF in food quality, establish RF as the superior modeling approach for spectroscopic prediction of quality parameters in crude palm oil, particularly when applied to optimally selected variable subsets using techniques such as GA, CARS, and UVE assessment^33,34^.

## Discussion

The multifaceted analysis revealed meaningful relationships between physicochemical properties, spectroscopic signatures, and predictive modeling of palm oil quality. Peroxide values ranged from 4.29 to 11.45 meq O₂/kg across samples, with most below the 10 meq/kg international threshold, indicating good oxidative stability^35^. Among the sampling locations, KAN exhibited the highest mean peroxide value (8.23 ± 1.65 meq O₂/kg), while MAK showed the most significant variability (CV = 0.307), suggesting local processing or storage differences. Iodine values (40.89–48.69 g I₂/100 g) were consistently lower than the standard reference range, suggesting a distinctive regional fatty acid composition rather than adulteration, which aligns with earlier findings by MacArthur et al.^3^. These observations provide a strong basis for linking the underlying chemical composition of the oils with their corresponding spectroscopic features.

Raman spectral analysis identified distinctive peaks following baseline correction and normalization at 1156–1657 cm^−1^ (C–H bending and C = C stretching), associated with unsaturation levels, and at 1748–1840 cm^−1^, reflecting oxidation status. These spectral signatures were consistent with prior studies^16,36^. The application of second-derivative preprocessing significantly enhanced model performance by resolving overlapping bands and minimizing baseline effects, yielding stronger correlations than other preprocessing techniques (e.g., 1st Der, WD, SG). This outcome supports the effectiveness of derivative-based preprocessing for heterogeneous food matrices, as emphasized by Haruna et al.^9^. Furthermore, variable selection techniques improved model efficiency by reducing spectral dimensionality while retaining critical information. Specifically, UVE-PLS retained only 4.4–6.4% of variables, while GA-PLS, guided by evolutionary principles, utilized 13.7–14.5%, demonstrating that selective dimensionality reduction can meaningfully enhance prediction accuracy without information loss.

Machine learning models built on these optimized spectral inputs showed consistent, quantitative performance. Random Forest (RF) methods outperformed Support Vector Machines (SVM), with GA-RF demonstrating the best results for both peroxide and iodine value predictions. As summarized in Table 3, the reliability hierarchy based on RPD followed the order: GA-RF > Standard RF > CARS-RF > UVE-RF > Standard PLS > UVE-PLS > GA-SVM > GA-PLS > CARS-PLS > Standard SVM > CARS-SVM > UVE-SVM. A similar sequence was observed for iodine value prediction, confirming the robustness of RF-based approaches. The superiority of GA-RF highlights the synergistic effects between the genetic algorithm’s efficient variable selection and Random Forest’s ensemble learning capacity—an interaction consistent with optimization principles described by Adugna et al.^25^. Beyond PV and IV, Raman spectroscopy is also sensitive to other palm oil quality attributes, including free fatty acids and carotenoids, indicating that extension of the present framework to multi-parameter prediction is feasible. While such an extension would require careful management of spectral overlap, response collinearity, and calibration data diversity, we recommend that future studies explicitly pursue simultaneous or parallel prediction of multiple quality indices to further enhance the diagnostic power and practical utility of Raman-based quality assessment in palm oil systems.

**Table 3:** Predictive performance of machine learning models with variable selection algorithms for peroxide and iodine value prediction in crude palm oil

Parameter	Machine Learning Models	Number of variables	Calibration set n = 133		Prediction set n = 67		RPD
			Rc	RMSEC	Rp	RMSEP	
Peroxide value (meq O_2_/kg)	SVM	1024	0.9796	0.3130	0.9439	0.8715	2.1877
	CARS- SVM	104	0.9799	0.2998	0.9584	0.8146	2.6223
	UVE- SVM	66	0.9801	0.3000	0.9736	0.7596	2.7392
	GA- SVM	148	0.9761	0.3272	0.9065	0.6531	3.0566
	RF	1024	0.9833	0.1459	0.9802	0.2735	6.2240
	CARS-RF	104	0.9916	0.1928	0.9599	0.4252	5.0331
	UVE-RF	66	0.9868	0.2418	0.9589	0.4304	4.9724
	GA-RF	148	0.9959	0.1346	0.9831	0.2765	7.7397
Iodine value (g I_2_/100 g)	SVM	1024	0.9807	0.3615	0.9559	0.9236	2.3871
	CARS- SVM	91	0.9789	0.3306	0.9424	0.9784	2.4697
	UVE- SVM	45	0.9598	0.4545	0.9125	0.6965	3.4467
	GA- SVM	140	0.9787	0.3367	0.9030	0.7103	2.9234
	RF	1024	0.9887	0.2393	0.9725	0.3748	6.0727
	CARS-RF	91	0.9888	0.2395	0.9475	0.5179	4.3962
	UVE-RF	45	0.9842	0.2842	0.9678	0.4058	5.6106
	GA-RF	140	0.9947	0.1638	0.9752	0.3561	6.3927

*n* number of samples, *Rc* Correlation Coefficient for the calibration set, *RMSEC* root mean square error of cross-validation *Rp* correlation coefficient for the predictionn set, *RMSEP* root mean square error of prediction, *RPD* residual predicted deviation, *SVM* support vector machine, *CARS-SVM* competitive adaptive reweighted sampling - support vector machine, *UVE-SVM* uninformative variable elimination - support vector machine, *GA-SVM* genetic algorithm - support vector machine, *RF* random forest, *CARS-RF* competitive adaptive reweighted sampling - random forest, *UVE-RF* uninformative variable elimination - random forest, *GA-RF* genetic algorithm - random forest.

When benchmarked against existing spectroscopic chemometric models for oil quality indices, the GA-RF–Raman approach developed in this work shows clearly superior predictive performance. Haruna et al. used FT‑NIR with CARS-PLS to estimate acid and peroxide values in crude peanut oil, reporting RPD values of 3.14–3.64, with Rp generally below 0.97 and RMSEP of 0.6–0.8 units^17^. Vinet et al. obtained RPD values of 3.52–4.20 and Rp up to approximately 0.98 for iodine and peroxide indices using MIR‑PLS, while their NIR models were limited to screening applications (RPD 2.18–2.68 with higher RMSEP)^32^. Liu et al. reported RPD values of 3.51–4.05, Rp below 0.98, and RMSEP typically above 0.5 when using SPA–PLS with FT‑IR and Raman data fusion for peroxide and acid values in edible oils^31^. In contrast, our optimized GA-RF models, based solely on second‑derivative Raman spectra of crude palm oil, achieved Rp values of 0.9831 (PV) and 0.9752 (IV), low prediction errors (RMSEP = 0.2765 and 0.3561, respectively), and high RPD values of 7.74 and 6.39. These results substantially exceed the analytical‑grade threshold of 3.0 and clearly surpass the performance of the above NIR, MIR, and multimodal IR–Raman fusion models. Although direct numerical comparison across studies should be interpreted with caution due to differences in oil matrices, index definitions, and validation protocols, our findings demonstrate that a single Raman measurement can provide highly robust predictions of oxidative and unsaturation indices in chemically complex crude oil systems.

Overall, this study demonstrates the practical effectiveness of integrating Raman spectroscopy with machine learning for rapid, non-destructive assessment of crude palm oil quality. By developing the models on samples sourced from Ghanaian open markets, the framework was intentionally grounded in market-representative variability, ensuring relevance to real-world production and distribution contexts. The resulting predictive models provide a reliable basis for simultaneously estimating peroxide and iodine values from a single spectral measurement, enabling efficient quality screening without reliance on wet-chemical assays. Compared with conventional analytical approaches, this spectroscopic–machine learning framework enables faster analysis, preserves sample integrity, and eliminates the need for chemical reagents, which is particularly advantageous for routine monitoring in resource-limited settings. Building on this foundation, extending the established framework to additional geographical regions, processing systems, and complementary quality attributes represents a logical next step toward broader application across the palm oil value chain.

## Methods

### Materials, reagents, and apparatus

All chemicals and reagents used in this study were of analytical grade. Acetic acid (≥99.5% purity), chloroform (≥99.0%), cyclohexane (≥99.0%), and Wijs iodine solution were purchased from Macklin Biochemical Co., Ltd. (Shanghai, China). Potassium iodide (≥99.0%), sodium thiosulfate solution, soluble starch, and potassium dichromate (dried at 110 °C) were obtained from Sinopharm Chemical Reagent Co., Ltd. (Shanghai, China). All solutions were prepared using ultrapure water (18.2 MΩ·cm) from a Milli-Q water purification system (Millipore Corp., Billerica, MA, USA).

### Sample acquisition

The crude palm oil samples were obtained from five major local markets in the capital city of Ghana: Agbogbloshie (AGB), Dome (DOM), Kaneshie (KAN), Madina (MAD), and Makola (MAK). These open markets typically aggregate crude palm oil produced by multiple small-scale and semi-mechanized processors, thereby reflecting a range of production practices and post-processing handling conditions encountered in real-world supply chains. From each market, 50 independent samples were purchased from different vendors to capture the natural variability in oil quality and processing-related differences, resulting in 200 samples. As is common for market-sourced crude palm oil, detailed information on palm fruit cultivar, specific processing parameters, and harvest season was not available. Each sample was collected in a dark glass bottle, transported under ambient conditions, and stored at 4°C before spectral and chemical analyses.

### Determination of peroxide value

The peroxide value (PV) determination was conducted following the AOAC 965.33 method. Approximately 5.00 ± 0.05 g of crude palm oil was weighed into a 250 mL glass-stoppered Erlenmeyer flask. To this sample, 30 mL of acetic acid-chloroform solution was added using a graduated cylinder, and the flask was swirled until the sample dissolved completely. Subsequently, 0.5 mL saturated potassium iodide solution was added. The flask was immediately stoppered and swirled for 1 min. Next, 30 mL of deionized water was added, and the flask was vigorously shaken to liberate iodine from the chloroform layer. The liberated iodine was titrated with 0.1 N sodium thiosulfate solution. After adding 1 mL of starch solution as an indicator, titration continued until the blue-gray color of the aqueous (upper) layer disappeared. The volume of titrant used was recorded to two decimal places. A blank titration was also conducted under the same conditions, using the same reagents but without the sample.

The peroxide value was calculated using the following formula:

1 $${\rm{Peroxide\; value}}({PV})\left({\rm{meq}}{{\rm{O}}}_{2}/{\rm{kg}}\right)=\frac{\left(S-B\right)\times N\times 1000}{W}$$

where S = sample titration volume (ml), B = blank titration volume (ml), N = normality of sodium thiosulfate, and W = sample weight (g). The peroxide value is expressed in milliequivalents of peroxide oxygen per kilogram of sample (meq O₂/kg).

### Determination of iodine value

The iodine value of crude palm oil was determined using the AOAC 993.20 Method (Wijs Cyclohexane-Acetic Acid Solvent Method). The CPO sample was first melted at 45 °C in a water bath and passed through a double layer of filter paper to remove solid impurities and trace moisture. After ensuring the sample was dry, 2.00 g of it was weighed into a clean, dry 500 mL glass-stoppered flask. Two blank determinations were prepared alongside the sample for accuracy. To the sample, 30 mL of cyclohexane-acetic acid solvent was added with swirling. Then, 50 mL of Wijs solution was dispensed into the flask, which was immediately stoppered and swirled to ensure thorough mixing. The flask was stored in the dark at 25 ± 5 °C for exactly 1 h. After the reaction period, 40 mL of 15% potassium iodide solution was added, followed immediately by 300 mL of distilled water. The liberated iodine was titrated with standardized 0.1 M sodium thiosulfate solution, with constant, vigorous shaking, until the yellow color had almost disappeared. Then, 1–2 mL of starch indicator solution was added, and titration continued until the blue color just disappeared. Blank determinations were conducted simultaneously under identical conditions. The iodine value was calculated using the equation:

2 $${\rm{Iodine\; value}}\left({\rm{IV}}\right)\left({{\rm{gI}}}_{2}/100{\rm{g}}\right)=\frac{(B-S)\times M\times 12.69}{W}$$

where B = titration volume for blank (mL), S = titration volume for sample (mL), M = molarity of the Na₂S₂O₃ solution (0.1 M), and W = weight of CPO sample.

### Raman spectra collection

Raman spectra were acquired using an RMS1000 spectrometer (Ocean Hood Opto-electronics Tech Co., Ltd, Shanghai, China) equipped with a ×10 visible objective lens, scanning from 500 to 2000 cm^−1^. The instrument used a 785 nm wavelength laser (150 mW) with a 100 μm spot. Before analysis, crude palm oil samples were melted at 45 °C, homogenized, and a thin film was placed on aluminum foil. Measurements employed a 600 grooves/mm grating with an integration time of 8 s and single scans. To ensure reproducibility, three spectra were recorded from different locations on each sample and averaged to obtain the final spectrum. Two sets of spectral data were collected for all 200 samples: one set was maintained as unprocessed raw data, while the second set underwent baseline correction and normalization. This approach enabled direct comparison between processed and unprocessed spectral features. All measurements were performed at controlled room temperature (25 ± 1 °C) and relative humidity (45%) to minimize environmental effects on the spectral features.

### Spectral partitioning and model evaluation

The dataset of 200 unprocessed Raman spectra was partitioned into calibration and prediction sets using a 2:1 ratio, yielding 133 samples for model development and 67 for validatio^37^. Model performance was rigorously assessed through multiple statistical metrics, including Root Mean Square Error for Calibration (RMSEC) and Prediction (RMSEP), Coefficient of Determination for Calibration (Rc) and Prediction (Rp), and Residual Predictive Deviation (RPD). High Rc and Rp values indicate strong internal correlations, while low RMSEC and RMSEP indicate greater predictive precision. Minimal disparities between calibration and prediction metrics suggest robust model stability. The Residual Predictive Deviation (RPD) provides critical performance insights: RPD values ≥ 3 indicate exceptional model reliability and accuracy, values between 2 and 3 indicate acceptable performance, and values below 2 indicate potential predictive limitations^38^.

### Theory of preprocessing tools

Spectroscopic data preprocessing is a critical initial stage in analytical methodologies, transforming raw spectral information into more interpretable and robust datasets. These preprocessing techniques are mathematical transformations that enhance signal quality, extract meaningful features, and improve the predictive accuracy of analytical models. By systematically refining spectral data, researchers can uncover chemical information that is obscured in raw spectra^39^. This study utilized first-derivative and second-derivative transformations, Wavelet Denoising (WD), and Savitzky-Golay (SG) smoothing to optimize spectral analysis.

The first-derivative transformation highlights rapid changes in spectral data, such as peaks or inflection points, while reducing baseline shifts. Calculating the rate of change between consecutive data points enhances the clarity of overlapping peaks and resolves subtle spectral features^40^. However, while it sharpens features, it can also amplify noise, requiring complementary preprocessing steps to mitigate this effect. The second-derivative transformation further refines spectral analysis by applying a double differentiation, amplifying sensitivity and resolving closely spaced peaks. This method eliminates broader baseline trends and emphasizes local spectral curvature, making it especially valuable in distinguishing overlapping spectral components in complex datasets^41^.

Wavelet Denoising (WD) and Savitzky-Golay (SG) smoothing complement these transformations by improving the signal-to-noise ratio. WD employs multi-resolution analysis to decompose spectral signals into approximation and detail coefficients, selectively suppressing noise while preserving critical spectral features^42^. This is particularly effective for Raman spectra, where sharp peaks hold vital information. SG smoothing, on the other hand, fits polynomials to local data segments within a moving window, filtering out high-frequency noise while retaining spectral integrity^43^. Together, these preprocessing methods provide essential tools for enhancing spectral quality and preserving critical chemical information in analytical methodologies.

### Theory of variable selection algorithms

Variable selection algorithms are essential analytical techniques in modern data science and machine learning, where identifying the most informative features is critical. These sophisticated methodologies employ distinct strategies to reduce data complexity, enhancing model performance, and extracting meaningful insights by systematically identifying and prioritizing the most relevant variables. In spectroscopic and chemometric applications, where datasets often contain numerous potentially correlated variables, these algorithms play a critical role in transforming raw spectral information into actionable predictive models^39^.

Uninformative Variable Elimination (UVE) operates on the principle of correlation-based feature screening, systematically evaluating the relationship between individual variables and target properties using statistical techniques to quantify each feature’s predictive potential. UVE fundamentally differs from traditional methods by focusing on intrinsic information content rather than relying solely on statistical significance^44^. By progressively eliminating variables with low correlation, UVE creates a more parsimonious dataset that captures essential spectral characteristics while minimizing noise and redundancy. In contrast, CARS represents a more advanced evolutionary approach, incorporating adaptive sampling and iterative refinement strategies with a dynamic weighting mechanism that continuously adjusts variable importance based on predictive performance. CARS employs an in whichhisticated sampling strategy where variables are selected probabilistically, with weights based on their correlation to the target property^45^. The method uses an exponential-decay function to progressively narrow the variable pool, creating a competitive environment in which only the most informative features survive multiple iterative evaluations.

The Genetic Algorithm (GA) introduces a biomimetic approach to variable selection, inspired by natural evolutionary processes. By representing potential variable combinations as “chromosomes” and applying principles of selection, crossover, and mutation, GA explores complex feature spaces with remarkable efficiency. Unlike traditional optimization techniques, GA can simultaneously evaluate multiple potential solutions, making it particularly powerful in handling non-linear and interaction-based relationships between variables^46^. The algorithm’s strength lies in its ability to navigate vast combinatorial spaces, discovering variable subsets that might be overlooked by more deterministic selection methods, offering a robust and flexible approach to feature selection across diverse analytical domains.

### Theory of support vector regression (SVR)

Support Vector Regression (SVR) is a supervised machine learning method derived from Support Vector Machines (SVMs) for predicting continuous numerical values. Unlike traditional regression methods that minimize the error between predicted and actual values, SVR introduces an epsilon-insensitive margin, allowing slight deviations without considering them errors. The key idea is to approximate the relationship between input features and outputs with minimal error while maintaining a tolerance margin^47^. For Raman spectroscopic data analysis, we employed the epsilon-SVR approach within the LIBSVM framework to handle spectral complexity. The Radial Basis Function (RBF) kernel was chosen for its ability to capture non-linear relationships without restrictive assumptions about spectral structures^48^.

Our methodology incorporated comprehensive preprocessing, including normalizing spectral intensities to the [−1, 1] range and applying Principal Component Analysis to preserve 95% of spectral variance while enhancing computational efficiency. Parameter optimization evolved from traditional grid searches to advanced computational strategies, integrating genetic algorithms and particle swarm optimization to efficiently explore the complex parameter space^43^. Rigorous cross-validation protocols ensured model selection based on generalization performance rather than spectral artifacts, and comparative analyses revealed that evolutionary optimization approaches consistently required fewer computational iterations to achieve comparable or superior predictive outcomes.

### Theory of random forest (RF)

Random Forest (RF) is an ensemble learning method that constructs multiple decision trees during training and outputs predictions by averaging their predictions^26^. Each tree in the forest is trained on a bootstrap sample of the training data, introducing variability that reduces the risk of overfitting commonly associated with single decision trees. In this study, 250 decision trees and a minimum leaf size of 10 were configured based on preliminary optimization trials and consistent with prior spectroscopic modeling studies, ensuring a balance between computational efficiency, model generalization, and predictive accuracy. The algorithm’s bootstrap sampling and random feature selection at splits created diverse trees for robust predictions, while the averaging process across all trees minimized variance and improved prediction stability. The RF approach proved particularly valuable for capturing non-linear relationships between Raman spectral features and quality parameters without requiring explicit feature transformations^49^.

Our RF implementation offers significant advantages for Raman spectroscopy. Its ability to model non-linear relationships makes it ideal for analyzing complex vibrational data where peak intensities correlate non-linearly with analyte concentrations^50^. Feature importance metrics enhance interpretability by identifying diagnostic Raman shifts linked to molecular vibrations. Additionally, RF is robust against baseline variations, peak shifts, and noise, making it suitable for real-world Raman applications with heterogeneous samples and instrumental variations^51^.

### Quantitative modeling strategy

Quantitative modeling of peroxide value (PV) and iodine value (IV) in crude palm oil was based on Raman spectral data. The modeling incorporated systematic preprocessing methods, including first- and second-derivative transformations, denoising techniques, and Savitzky-Golay (SG) smoothing. These strategies enhanced spectral information quality and minimized noise interference. The most effective preprocessing method was selected as input for advanced variable selection algorithms: Competitive Adaptive Reweighted Sampling (CARS), Genetic Algorithm (GA), and *Uninformative Variable Elimination (UVE)*.

We implemented a comprehensive analytical framework comprising 12 distinct predictive models that evaluated variable selection techniques with diverse regression algorithms. Three foundational regression algorithms—PLS, SVM, and RF—were implemented both independently and in combination with CARS, GA, and UVE, yielding nine variable selection-regression algorithm combinations plus three full-spectrum baseline models. Each model underwent rigorous parameter optimization, including latent-variable selection for PLS, kernel-function parametrization for SVM, and hyperparameter tuning for RF. By incorporating both linear (PLS) and non-linear (SVM, RF) regression approaches, the study addressed complex spectral-chemical relationships in Raman spectroscopic data^52^.

### Statistical analysis

**Fig. 4:**
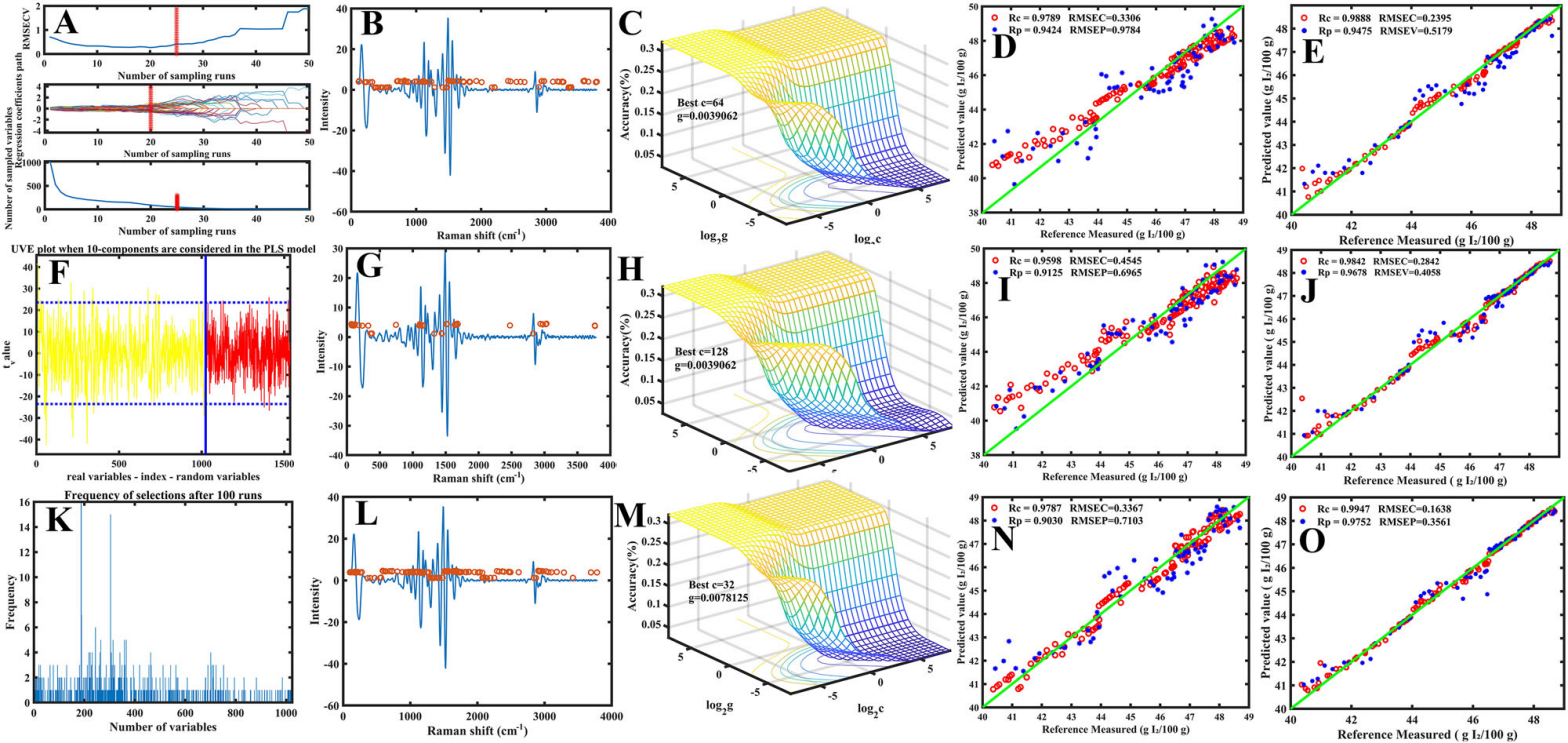
Results of variable selection algorithms and machine learning models for iodine value (IV) prediction in crude palm oil using Raman spectroscopy. CARS variable selection results: **A** variable selection process showing RMSECV values and the number of retained variables, **B** distribution of selected variables in the full Raman spectrum, **C** 3D response surface for SVM hyperparameter optimization, **D** scatter plot of CARS-SVM model predictions versus reference values, **E** scatter plot of CARS-RF model predictions versus reference values. UVE variable selection results: **F** reliability plot distinguishing relevant variables (yellow) from random variables (red), **G** distribution of selected variables, **H** 3D response surface for SVM hyperparameter optimization, **I** UVE-SVM prediction scatter plot, **J** UVE-RF prediction scatter plot. GA variable selection results: **K** frequency distribution of selected variables, **L** distribution of selected variables in the spectrum, **M** 3D response surface for SVM hyperparameter optimization, **N** GA-SVM prediction scatter plot, **O** GA-RF prediction scatter plot. The selected variables for iodine value modeling are predominantly located in Raman bands linked to degree of unsaturation, especially within 1287–1657 cm^−1^ corresponding to C–H bending and C=C stretching vibrations, confirming their chemical relevance for IV prediction.

All samples were analyzed in triplicate, and results were reported as means ± standard deviations. Statistical analysis was performed using OriginLab (Version 2019, Microcal Inc., Northampton, MA, USA), employing one-way analysis of variance (ANOVA) followed by Tukey’s test to assess significant differences in mean values at p < 0.05. Raman spectral data analysis and modeling were conducted using MATLAB version R2022a (MathWorks, Natick, USA) on a computer equipped with an Intel(R) Core(TM) i7-6700T CPU @ 2.80 GHz (2.81 GHz), 15.9 GB RAM, running Windows 10 Pro 64-bit. Figure 4 presents a schematic illustration of the entire research process.

## Supplementary information

Supplementary Information

## Data Availability

The datasets generated and analyzed during the current study are available from the corresponding author upon reasonable request.

## Code availability

Custom scripts used for data analysis are available from the corresponding author upon reasonable request.
