# Supplementary material for: Machine learning-assisted Raman spectroscopy for non-destructive analysis of crude palm oil quality
Source: NPJ Sci Food. 2026 Jan 14;10:41. doi: 10.1038/s41538-025-00688-1 (PMC12887024; doi:10.1038/s41538-025-00688-1)
Supplement: Supplementary file 1 — Supplementary Information [file 41538_2025_688_MOESM1_ESM.pdf]

Supplementary Material

Machine Learning-Assisted Raman Spectroscopy for Non-Destructive Analysis of Crude Palm Oil Quality

Selorm Yao-Say Solomon Adade[a,c,d], Akwasi Akomeah Agyekum[e], Xorlali Nunekpeku[b], Nana Adwoa Nkuma Johnson[a,c], John-Nelson Ekumah[b,c], Bridget Ama Kwadzokpui[b], Hao Lin[b], Huanhuan Li[b], Quansheng Chen[a]*

[a]College of Ocean Food and Biological Engineering, Jimei University, Xiamen 361021, PR China

[b]School of Food and Biological Engineering, Jiangsu University, Zhenjiang 212013, P. R. China

[c]Centre for Agribusiness Development and Mechanization in Africa (CADMA AgriSolutions), Ho, 00233, Ghana

[d]Department of Nutrition and Dietetics, Ho Teaching Hospital, P. O. Box MA 374, Ho, Ghana

[e]Nutrition Research Centre, Ghana Atomic Energy Commission, Kwabenya, Accra, 00233, Ghana

[f] School of Agriculture, Department of Agricultural Engineering, University of Cape Coast, 00233, Ghana

*Corresponding author

E-mail addresses: chenqs@jmu.edu.cn (Q. Chen)

Table S1. Key Raman spectral markers for IV and PV determination in crude palm oil

Peak Position (cm^{-1})	Molecular Assignment	References
1287	=C–H bending (cis), associated with unsaturated bonds (IV)	(Berghian-Grosan & Magdas, 2020; Mohamed Dafaa Allah Banaga et al., 2021)
1442	CH_2 bending (scissoring), deformation oxidation marker (PV)	(Berghian-Grosan & Magdas, 2020; Mohamed Dafaa Allah Banaga et al., 2021; Vaskova & Buckova, 2018)
1602	C=C stretching (cis), strongly linked to unsaturation (IV)	(Berghian-Grosan & Magdas, 2020; Vaskova & Buckova, 2018)
1657	C=C stretching from cis double bonds (IV)	(Berghian-Grosan & Magdas, 2020; Mohamed Dafaa Allah Banaga et al., 2021)
1748	C=O stretching (esters and peroxides), oxidation marker (PV)	(Qiu et al., 2019; Vaskova & Buckova, 2018)

Table S2. Performance of various pretreatment algorithms for peroxide and iodine value prediction in palm oil samples

**Parameter**	**Pretreatment Algorithm**	**Principal Component**	**Calibration set n=133**		**Prediction set n=67**		**RPD**
			**Rc**	**RMSEC**	**Rp**	**RMSEP**	
Peroxide value	Raw	7	0.9562	0.6178	0.9249	0.6175	2.7880
	1st Der	6	0.9441	0.4768	0.9359	0.6332	3.2473
	**2nd Der**	**6**	**0.9597**	**0.5154**	**0.9481**	**0.6367**	**4.8935**
	Denoise	7	0.9390	0.4297	0.9237	0.4911	3.0606
	SG	7	0.9459	0.4599	0.9133	0.4981	3.5749
Iodine value	Raw	7	0.9469	0.3645	0.9377	0.6712	3.4346
	1st Der	6	0.9362	0.5021	0.9286	0.8195	2.7613
	**2nd Der**	**6**	**0.9692**	**0.3565**	**0.9400**	**0.5002**	**3.9139**
	Denoise	7	0.9546	0.3953	0.9325	0.5951	3.8336
	SG	7	0.9426	0.4189	0.9327	0.5354	3.2099

n = number of samples, Rc = Correlation Coefficient for the calibration set, RMSEC = Root Mean Square Error of Cross Validation, Rp = Correlation Coefficient for the prediction set, RMSEP = Root Mean Square Error of Prediction, RPD = Residual Predicted Deviation, 1st Der = First Derivative, 2nd Der = Second Derivative, SG = Savitzky-Golay.
